# MYC is not detected in highly proliferating normal spermatogonia but is coupled with CIP2A in testicular cancers

**DOI:** 10.19185/matters.201602000040

**Published:** 2016-03-03

**Authors:** Sami Ventelä, Juho-Antti Mäkelä, Rosalie C Sears, Jorma Toppari, Jukka Westermarck

**Affiliations:** Department of Physiology, University of Turku; Department of Molecular and Medical Genetics and Knight Cancer Institute, Oregon Health and Science University; Centre for Biotechnology and Department of Pathology, University of Turku

## Abstract

High MYC expression is linked to proliferative activity in most normal tissues and in cancer. MYC also supports self-renewal and proliferation of many types of tissue progenitor cells. Cancerous inhibitor of PP2A (CIP2A) promotes MYC phosphorylation and activity during intestinal crypt regeneration *in vivo* and in various cancers. CIP2A also supports male germ cell proliferation *in vivo*. However, the role of MYC in normal germ cell proliferation and spermatogonial progenitor self-renewal is currently unclear. Here, we demonstrate that male germ cells are CIP2A-positive but lack detectable levels of MYC protein; whereas MYC is highly expressed in Leydig cells and peritubular myoid cells contributing thereby to the testicular stem cell niche. On the other hand, MYC was co-expressed with CIP2A in testicular cancers. These results demonstrate that CIP2A and MYC are spatially uncoupled in the regulation of spermatogenesis, but functional relationship between these two human oncoproteins is established during testicular cancer transformation. We propose that further analysis of mechanisms of MYC silencing in spermatogonial progenitors may reveal novel fundamental information relevant to understanding of MYC expression in cancer.

## Introduction

Animal reproduction is entirely dependent on adult germ cell proliferation. A special mode of proliferation, the self-renewal of tissue-specific stem - and progenitor cells is required both to support the renewal of tissues and for the rapid production of sperm. Spermatogonia, that reside in the outer basal layer of the seminiferous tubules, are among the most actively dividing cells in human body and therefore characterizing regulators of their self-renewal could reveal generally important insights into the biology of progenitor and stem cells. Moreover, comparative analysis of molecular mechanisms driving testicular proliferation and self-renewal involved in normal spermatogenesis but also in the context of testicular cancers could reveal causal mechanisms generally relevant for cellular transformation.

Protein phosphatase 2A (PP2A) accounts for a large fraction of the total cellular serine/threonine phosphatase activity (Lambrecht 2013^[1]^). A PP2A-interacting protein designated cancerous inhibitor of PP2A (CIP2A) inhibits degradation of serine 62 phosphorylated form of the oncoprotein MYC (Myant 2015^[2]^). Physiologically, CIP2A promotes spermatogenesis by supporting maintenance of spermatogonial progenitor pool (Ventel _2012^[3]^), and facilitates intestinal crypt progenitor-driven intestinal regeneration *in vivo* (Myant 2015[2]). However, whereas the latter was shown to involve CIP2A-mediated regulation of MYC (Myant 2015[2]), the expression and functional role for MYC in spermatogonial progenitors is poorly understood.

## Objective

To study whether the spermatogonia, that are among the most actively dividing cells in human body, and exhibit strong self-renewal capacity, express MYC. To establish, whether CIP2A-mediated MYC regulation observed in other highly proliferating cell types exists also in spermatogonia.

## Results & Discussion

**Figure 1:**
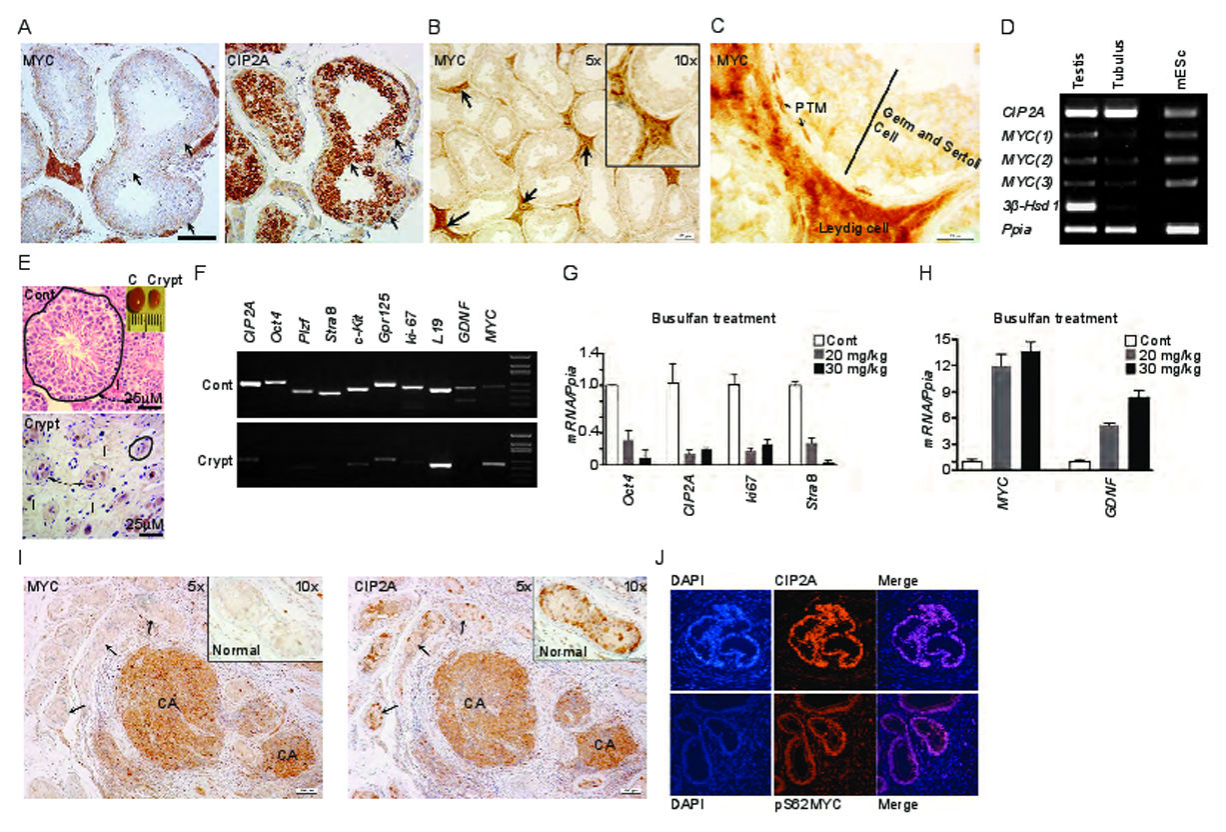
MYC and CIP2A are spatially uncoupled in the normal testis but co-expressed in testicular cancer. (A) MYC and CIP2A immunohistochemistry of the adult human testis showed the deficiency of MYC protein expression in male germ cells containing strong CIP2A protein expression (arrows). (B) Intensive MYC staining in the interstitial tissue (arrows). Insert: Interstitial tissue Leydig cells are strongly MYC positive, whereas the germ cells and the Sertoli cells are MYC negative. (C) The only cells within the seminiferous tubules that were MYC positive were the peritubular myoid cells (PTM). (D) RT-PCR analyses of indicated genes from the mouse whole testis lysate (Testis), isolated seminiferous tubules (Tubulus) and mouse embryonic stem cells (mESc). MYC expression was detected with three independent MYC specific primers. The purity of seminiferous tubuli extraction was demonstrated by the lack of Leydig cell derived 3b-Hsd1 transcript. Ppia (cyclophilin A) was used as house-keeping control gene. (E) Operatively induced cryptorchidism (Crypt) reduced the size of the testis (insert). Histology of the testis was altered (larger image), the size of the seminiferous tubules decreased (arrows and black circle), and the relative amount of the interstitial tissue (I) was increased in the operated testis. Representative data from the mouse after 6 months of cryptorchid operation. Black bars: 25 μm. (F) RT-PCR analyses of the cryptorchid testis showed downregulation of spermatogonia specific markers (CIP2A, Oct4, Plzf, Stra8, c-Kit, Gpr125) as well as ki-67 and Sertoli cell specific marker GDNF, when compared to the unoperated control testis of the same mouse. MYC expression instead increased in the cryptorchid testis as compared to control testis. L19 was used as an internal house-keeping control gene. (G and H) Intraperitoneally injected busulfan specifically depleted male germ cells. qRT-PCR analyses showed dose dependent downregulation of ki-67 and CIP2A and an increase of MYC expression 4 weeks after busulfan injection. (I) MYC and CIP2A staining from representative samples of human testicular cancer. Normal seminiferous tubules in cancer samples (arrows) do not express MYC whereas their adjacent cancerous (CA) lesions are highly MYC positive. CIP2A stains positively in both tissue types. Positive immunodetection of MYC in cancerous lesions confirms efficient MYC immunoepitope retrieval and antibody function. (J) Testicular cancer sample with expression of serine-62 phosphorylated MYC (S62-MYC) and CIP2A.

Based on (a) expression and functional role of CIP2A in spermatogonia (Ventel _2012^[3]^), (b) the established functional link between CIP2A and MYC (Myant 2015^[2]^), and (c) the role of both CIP2A and MYC in regulating progenitor cell fate (Ventel _2012[3])(Myant 2015[2])(Wilkins 2008^[4]^), we assessed the status of MYC in spermatogonial progenitor cells, spermatogonia. Systemic inhibition of MYC induced dramatic testicular atrophy marked by a loss of spermatogonia and spermatocytes (Soucek 2008^[5]^). However, to our knowledge MYC protein expression has not been demonstrated from testicular tissue as yet, and thus it has been unclear which testicular cell types express MYC. This is especially the case in the light of earlier results that mouse germ cells appear to proliferate in the absence of MYC transcription (Stewart 1984^[6]^). Therefore we analysed human testis samples by immunohistochemistry. While the testicular interstitial tissue was strongly immunopositive for MYC, its expression was not detected in the germ cells which were CIP2A positive (Figure 1A, B). Instead the weak positive MYC staining in the seminiferous tubules derived from the peritubular myoid cells (PTMs) (Figure 1C).

Unfortunately none of the tested MYC antibodies revealed a specific staining pattern in the mouse testis (data not shown). Therefore, to clarify MYC expression pattern in the mouse testis, we performed RT-PCR analyses using three different primers to amplify MYC mRNAs in the whole testis samples and dissected seminiferous tubules (Figure 1D). Murine embryonic stem cells were used as a positive control for MYC. CIP2A, which is a spermatogonially expressed gene, showed equal expression in the whole testis samples and isolated seminiferous tubules. MYC mRNA on the other hand was detected in the whole testis samples that were also positive for the interstitial tissue-originating Leydig cell-specific marker 3β-Hsd1. Dissected seminiferous tubules instead showed very low MYC expression (apparently from peritubular myoid cells) and almost no detectable 3β-Hsd1, confirming the purity of tubule isolation (Figure 1D). These results support the IHC staining results of human testis samples and suggest that faint MYC mRNA expression detected in dissected seminiferous tubules originate from PTM cells that surround the tubules. To experimentally validate the spatial uncoupling of CIP2A and MYC in the normal testis, we used an induced cryptorchidism mouse model where one testis was operatively placed in the intraperitoneal cavity, causing spermatogenesis arrest by preventing proliferation of spermatogonia. Induced cryptorchidism caused a significant reduction in the size of the operated testis (Fig. 1E, insert (Crypt)). Histologically, the size of seminiferous tubules was greatly diminished (Fig. 1E, black circle) whereas relative volume of interstitial space was greatly increased (Fig. 1D(I)). Gene expression analysis of control and cryptorchid testes demonstrated that expression of spermatogonial markers (*CIP2A*, *Oct4*, *Plzf*, *Stra8*, *c-Kit*, *Gpr125*) as well as proliferation (*ki-67*) was reduced in the cryptorchid testis as compared to the house-keeping gene L19 (Figure 1F). However, opposite to all other markers, and concomitant with the relative increase in somatic cells and testicular interstitial space, expression of MYC and Sertoli cell specific marker GDNF were increased in the cryptorchid testis as compared to the control testis (Figure 1F). Similar results were obtained when mice were treated with an alkylating chemotherapy agent busulfan, which selectively kills proliferating male germ cells causing similar spermatogenetic arrest and relative increase in interstitial tissue and Sertoli cells as cryptorchidism (Fig. 1G and H). These results provide indirect evidence that MYC is not expressed in highly proliferating spermatogonia either in the mouse or human. Consequently, CIP2A’s role in promoting spermatogenesis is unlikely to depend on its ability to stabilize MYC.

These results identified spermatogonia as a rare example of a highly proliferative normal cell type that does not express detectable levels of MYC. To determine whether this is true also in testicular germ cell cancers, we examined CIP2A and MYC protein expression levels by immunohistochemistry in seminoma and embryonic carcinoma samples. Nineteen out of twenty TC samples showed expression of CIP2A and 18/20 expressed also MYC (Supplementary Table 1). In some samples, normal seminiferous tubules could still be observed (see arrows and inserts in Fig. 1I), which allowed us to compare the expression of CIP2A and MYC between non-malignant and malignant areas on the same tissue section. Consistent with all other data, MYC was not expressed in normal seminiferous tubules, while the antibody detected clear MYC expression in the adjacent cancer lesion (Fig. 1I, and supplementary Fig. 1(CA)). CIP2A was expressed in both normal and cancerous tissues (Fig. 1I and supplementary Fig. 1). Moreover, in cancerous tissue, MYC was phosphorylated at serine 62 (Fig. 1J), which is consistent with CIP2A’s functional role in supporting expression of serine 62 phosphorylated MYC both in cancer (Junttila 2007^[7]^) and during intestinal regeneration *in vivo* (Myant 2015[2]).

Our results strongly indicate that CIP2A promotes spermatogenesis by a mechanism different from its established role of stabilizing MYC in cancer cells. Also, our results are interesting in the light of previous findings that another MYC family gene, MYCN, was shown to be expressed in isolated spermatogonial cells *in vitro* (Braydich Stolle_2007^[8]^) suggesting that spermatogonial proliferation is another cellular system in which MYCN can compensate for lack of MYC expression. Interestingly, recent studies have shown that in addition to MYC, CIP2A inhibits PP2A activity towards AKT kinase (Chen 2010^[9]^). Taking into account the reported role for AKT in promoting self-renewal of isolated spermatogonial stem and progenitor cells (Lee 2007^[10]^), CIP2A may also exert its effects on spermatogenesis via regulating AKT activity. In addition to the relevance of the present results for understanding CIP2A’s role in spermatogenesis, these findings reveal novel insights into MYC biology. Our results that are based on carefully controlled IHC protocol demonstrate that MYC is not expressed in highly proliferating male germ cells with self-renewal capacity. These results agree with those of mRNA analysis of MYC expression in mouse germ cells (Stewart 1984[6]). Our results identify testicular interstitial Leydig cells and peritubular myoid cells as the main sources of MYC expression in the testis. These cell types contribute to the establishment of the testicular stem cell niche (Oatley 2008^[11]^). Therefore, our results also suggest a potential role for MYC in the formation of the testicular stem cell niche.

## Conclusions

These results demonstrate that CIP2A and MYC are spatially uncoupled in the regulation of spermatogenesis, but functional relationship between these two human oncoproteins is established during transformation to testicular cancer.

## Limitations

Lack of reliable detection of MYC protein by immunohistochemistry in the mouse testis is a limitation of this study. However, our conclusions that MYC is not expressed by mouse germ cells is strongly supported both by the study by Stewart et al., (Stewart 1984^[6]^) and by three different approaches to demonstrate a lack of correlation between MYC mRNA expression and markers of self-renewal and proliferation in spermatogonia (Fig. 1D–H).

## Conjectures

Lack of detectable MYC expression in spermatogonial progenitors is an intriguing finding because MYC is prototypically expressed in highly proliferative normal and malignant cells. Based on our and published data (Stewart 1984^[6]^), MYC expression is silenced in normal SPCs but de-repressed in testicular cancer cells. Identifying the molecular mechanisms by which MYC expression is repressed during normal spermatogenesis may lend novel insights into MYC regulation in various physiological and pathological situations. On the other hand, we speculate that germ cell tumorigenesis may arise from the loss of germ line-specific inhibitors of MYC gene expression that in normal germ cells prevent excessive pluripotency and self-renewal, but when absent in abnormal germ cells, result in the conversion to germ cell cancer.

## Methods

### Immunohistochemistry and tissue samples

Formalin-fixed, paraffin embedded sections of mouse and human organs were cut into 6 μm thin sections, deparaffinized and thereafter rehydrated. Epitope retrieval was then performed in 10mM Tris-EDTA-buffer pH 9,0 during 4 min in microwave oven 850W followed by 15min 150W. After cooling down for 20 min at RT, samples were rinsed properly in water. Concerning the staining procedure, the slides were firstly blocked for 10 min in 3% BSA in PBS. After rinsing in Tris-HCl pH 7,4, incubation of primary antibody was done for 60 min in 3% BSA/PBS. Following few rinses, appropriate secondary antibody (Dako EnVision anti-rabbit or anti-mouse) incubation was done for 30 min. Slides, again rinsed, were then incubated in DAB+ liquid Dako (K3468) for 10 min, and then rinsed in water. Samples were incubated in Mayers HTX for 1 min, rinsed with tap water, and finally dehydrated, cleared and mounted. Human testicular samples were obtained from patient diagnosed testicular neoplasm and therefore underwent orchiectomy.

### Tissues sample homogenenization and RNA extraction

Liquid nitrogen frozen mouse samples were homogenized using the MagNA Lyser and MagNA Lyser Green Beads. Briefly, RNA and protein samples were homogeneized in respective lysing buffers, RA1 from Macherey Nagel for RNA; RIPA buffer (1X PBS; 1% Nonidet P-40; 0.5% sodium deoxycholate; 0.1% SDS) for protein samples. 1 to 4 cycles (6500 rpm, 50 sec) were used to homogeneize the tissues, an ice-cooling step of 2 min being done between each cycle. Total RNA was extracted and cleaned up from the lysate using the Nucleospin kit (Macherey Nagel), including a DNAse treatment step.

### Antibodies

The following antibodies were used for immunohistochemical stainings of paraffin embedded tissues: CIP2A: rabbit polyclonal anti-CIP2A (Hoo 2002^[11]^), MYC: mouse monoclonal 26 9E10 (sc-40, Santa Cruz Biotechnology) and S62-MYC: rabbit polyclonal anti-S62-MYC (Escamilla Powers_2006^[12]^).

### RT-PCR analysis

For cDNA synthesis 1 μg total RNA was incubated with 250 ng random hexamer for 5 min at 70°C, then cooled down on ice for another 5 min. Total RNA was reverse transcribed in a final volume of 25 μL containing enzyme buffer, 10 units of RNAse inhibitor, DTT, 0,5 mM deoxynucleotide triphosphate, and 5 units MMLV reverse transcriptase. The samples were incubated at room temperature for 10 min, then at 42°C for 50 min. The reverse transcriptase was finally inactivated by heating at 70°C for 15 min before PCR amplification. The quantification was based on the standard curve method. The data were normalized using β-actin. Oligonucleotides were obtained from Proligo. For quantitative real-time PCR, 2 μL of diluted reverse transcription reaction samples (1/10) were added to 8 μL of a PCR mixture made up of 5 μL of PCR Master Mix (Applied Biosystems), 1 μL of each primer at a concentration of 3 μM, and 1 μL of specific probe at a concentration of 2 μM. The thermal cycling conditions comprised an initial step at 50°C for 2 min and a denaturation step at 95°C for 10 min followed by 40 cycles at 95°C for 15 and 60°C for 1 min. All PCRs were carried out using an ABI Prism 7000 Sequence Detection System (Applied Biosystems). The specificity of each primer couple was shown by a dissociation curve analysis. Results are derived from the average of at least two independent experiments. Gene expression was reported relative to housekeeping gene.

### Surgically induced cryptorchidism and Busulfan injection

Surgical unilateral cryptorchidism (Crypt) of the right testes was induced in nine male mice (C57BL/6) at 8 weeks of age. Each mouse was anesthetized, and a lateral incision was made on the abdominal wall above the inguinal canal. The right testis was pulled away from the scrotum into the abdominal cavity. The inguinal canal was closed with nonabsorbable sutures. The mice were sacrificed after 6 months, and both testes were collected for histological and quantitative polymerase chain reaction (PCR) (qPCR) analyses.

Busulfan (Busilvex, Myleran, GlaxoSmithKline, United Kingdom) was dissolved in dimethyl sulfoxide and mixed with an equal volume of sterile distilled water before intraperitoneal injection into 8 weeks old C57BL/6 mice. Mice were treated with 20 or 30 mg/kg busulfan. Control mice were injected with 30 mg/kg dimethyl sulfoxide. Four weeks after injection the mice were sacrificed and testes were collected for histological and quantitative polymerase chain reaction (PCR) (qPCR) analyses.

## Supplementary Material

Supplementary Materials
