# Supplementary material for: MYC is not detected in highly proliferating normal spermatogonia but is coupled with CIP2A in testicular cancers
Source: Matters (Zur). Author manuscript; Available in PMC 2018 Mar 8. (PMC5843371; doi:10.19185/matters.201602000040)
Supplement: Supplementary Materials [file NIHMS859414-supplement-Supplementary_Materials.pdf]

Supplementary table 1. Expression of CIP2A and MYC in 20 different testicular cancer patient samples

| | CIP2A | MYC |
|---|---|---|
| *n* | 20 | 20 |
| Positive | 19/20 | 18/20 |
| % | 95 | 90 |

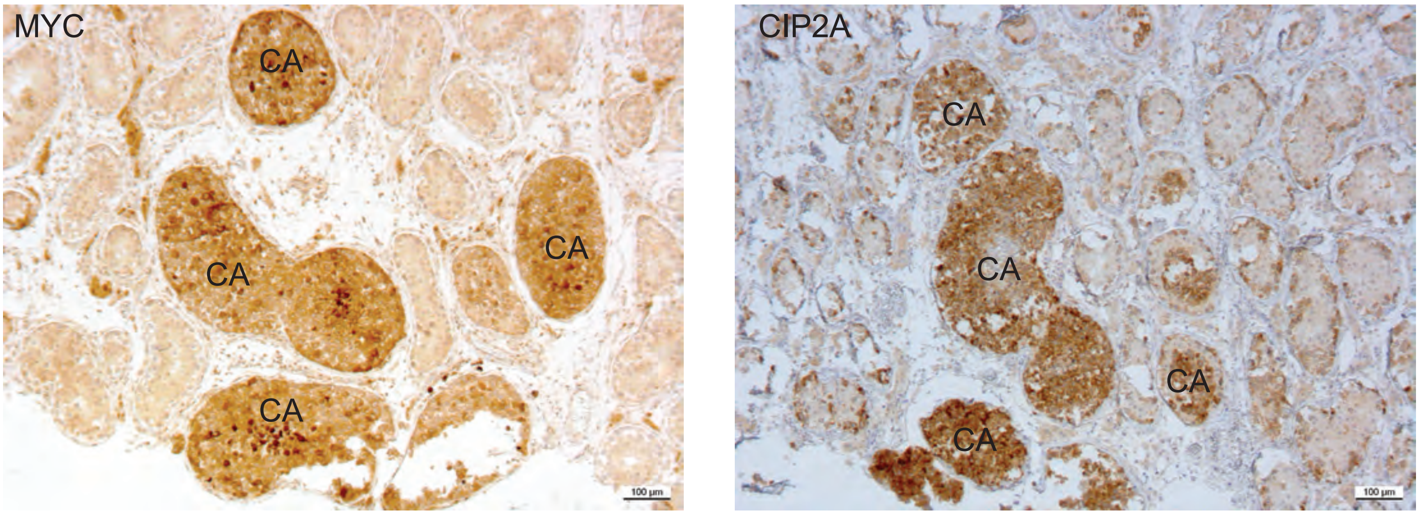


**Supplementary Fig. 1 Additional IHC image of co-expression of MYC and CIP2A in testicular cancer samples (CA) but lack of MYC expression in adjacent normal seminiferous tubules.**
